# Daily care demands of children with complex chronic conditions at home: a scoping review

**DOI:** 10.1590/0034-7167-2025-0341

**Published:** 2026-09-18

**Authors:** Tânia Barroso Barreto, Viviane Medeiros Machado, Mariana Setúbal Nassar de Carvalho, Carla Trevisan Martins Ribeiro

**Affiliations:** I Instituto Nacional da Saúde da Mulher da Criança e do Adolescente Fernandes Figueira/FIOCRUZ. Rio de Janeiro, Rio de Janeiro, Brazil

**Keywords:** Children with Disabilities, Chronic Disease, Home Care, Mechanical Ventilation, Caregivers., Niños con Discapacidad, Enfermedades Crónicas, Cuidado Domiciliario, Ventilación Mecánica, Cuidadores.

## Abstract

**Objectives:**

to describe the main home care needs of children with complex chronic conditions and technology dependencies after hospital discharge.

**Methods:**

a scoping review focusing on the care needed at home for children with complex chronic conditions and technology dependency. The search was conducted using the terms “child with disability”, “chronic disease”, “home care”, “mechanical ventilation”, and “caregivers”, as well as similar terms in English and Spanish, considering the last ten years.

**Results:**

seven studies were included. The main care areas identified were airway management, mechanical ventilation, life support equipment, ostomies, diet and medication administration, equipment cleaning, vital sign monitoring, basic life support, wound care, postural changes, personal hygiene, and bathing.

**Conclusions:**

the needs of children with complex chronic conditions at home require technical knowledge and adaptation of unique routines. This information contributes to the development of training programs for caregivers, with nurses acting as facilitators.

## RESUMEN

**Objetivos:**

describir las principales necesidades de atención domiciliaria de niños con enfermedades crónicas complejas y dependencia tecnológica tras el alta hospitalaria.

**Métodos:**

revisión exploratoria centrada en la atención domiciliaria necesaria para niños con enfermedades crónicas complejas y dependencia tecnológica. La búsqueda se realizó utilizando los términos “niño con discapacidad”, “enfermedad crónica”, “atención domiciliaria”, “ventilación mecánica” y “cuidadores”, así como términos similares en inglés y español, considerando los últimos diez años.

**Resultados:**

se incluyeron siete estudios. Las principales áreas de atención identificadas fueron el manejo de la vía aérea, la ventilación mecánica, los equipos de soporte vital, las ostomías, la administración de dieta y medicación, la limpieza de los equipos, la monitorización de constantes vitales, el soporte vital básico, el cuidado de heridas, los cambios posturales, la higiene personal y el baño.

**Conclusiones:**

las necesidades de los niños con enfermedades crónicas complejas en el hogar requieren conocimientos técnicos y la adaptación de rutinas específicas. Esta información contribuye al desarrollo de programas de capacitación para cuidadores, donde la enfermera actúa como facilitadora.

## INTRODUCTION

The epidemiological profile of hospitalized children in Brazil and worldwide has transformed in recent decades, with a significant increase in prolonged hospital stays, especially due to the increased survival rates of children with chronic health conditions and their long-term dependence on technology^(1)^. However, the prolonged stay of these children in hospital beds has a significant impact on the lack of beds in Intensive Care Units, the high cost of care, and consequences for child development and family unit maintenance, leading to the imminent need to discharge this population from hospitals^(2)^.

Complex chronic conditions (CCCs) can be defined as diseases that have permanent sequels and require care for long periods, often throughout individuals’ life cycle, in a model that must have access to life support technologies, such as non-invasive ventilation, tracheostomy and gastrostomy devices, among others^(3)^. It is understood, therefore, that chronic disease is a health condition with repercussions on different bodily structures and functions, which is usually not fatal, but which requires multiple and complex care^(2)^.

In Brazil, pediatric hospital programs and services that are a reference for comprehensive care for children with CCCs base their main organizational strategy on coordinating comprehensive care aimed at facilitating hospital discharge, as this has proven to have the best outcomes for the health and well-being of children and their families^(1)^. The discharge of this population from the hospital is often a challenge for healthcare teams, making planned and coordinated discharge essential, with mapping of the support network and the assistance network near patients’ homes, as well as training for daily care during the hospitalization period. It is important that caregivers learn about daily childcare, from correct positioning in bed/crib or the importance of handwashing to procedures such as suctioning, feeding, and dressing changes, as they will be responsible for these cares at home^(4)^.

Family caregiver training is becoming a valuable tool for the safe discharge of children and adolescents with CCCS. Even with the support of a multidisciplinary team with frequent home visits after discharge, as agreed upon with the community, the family plays a crucial role in providing the necessary care^(5)^. Since family caregivers need preparation during the hospital discharge process so that they can safely provide the necessary care to children at home, taking the lead in this care, a question arises: what care needs do children with CCCs require at home?

The Ministry of Health published a Practical Guide for Caregivers in 2008^(6)^, which aims to guide caregivers in providing healthcare to people of any age, bedridden or with physical limitations, who require special care at home. However, this guide, besides being outdated, is not specifically designed for children. Therefore, it is essential to search for international research and guidelines that provide information about the study’s objective: the home care needs of children with CCCs who are technology-dependent.

## OBJECTIVES

To describe the main home care needs of children with CCCs and technology dependencies after hospital discharge.

## METHODS

This is a scoping review, conducted from October 2024 to February 2025, and carried out based on the JBI Evidence Synthesis Manual guidelines^(7)^, following five stages: 1 - research question description; 2 - relevant study identification; 3 - study selection; 4 - data mapping; and 5 - result summary and report. The protocol for this review was registered in PROSPERO. (https://www.crd.york.ac.uk/PROSPERO/view/CRD420251044808) and in the Open Science Framework (https://doi.org/10.17605/OSF.IO/NXRK8). The review was conducted in accordance with the Preferred Reporting Items for Systematic Reviews and Meta-Analyses extension for Scoping Reviews (PRISMA-ScR)^(7-9)^.

### Scope review stages:

#### Stage 1 - Research question description

The first stage involved formulating a research question using the acronym PCC (Population (children with CCCs dependent on technology after the dehospitalization process), Concept (daily care needs), and Context (home care by caregivers/parents)), namely: what daily care is needed at home for children with CCCs dependent on technology after hospital discharge?

#### Stage 2 - Relevant study identification

The second stage involved selecting the databases and developing search strategies using descriptors and Boolean operators.

Controlled vocabulary descriptors were selected from the Health Sciences Descriptors, Medical Subject Headings, CINAHL, and Emtree/Embase. Bibliographic search was conducted from October to December 2024 in the US National Library of Medicine National Institutes of Health (PubMed Central), Latin American and Caribbean Literature in Health Sciences (LILACS), Web of Science, Embase, and Scientific Electronic Library Online (SciELO) databases (Chart 1).

**Chart 1:** Search Strategy with Descriptors, Rio de Janeiro, Rio de Janeiro, Brazil, 2025

MEDLINE via PubMed	(Disabled children) AND (Chronic disease) AND (Home nursing OR Home Care Services) AND (Respiration, artificial) AND (Oxygen Inhalation Therapy) AND
LILACS	(*Niños con Discapacidad*) AND (*Enfermedad Crónica*) AND (*Atención Domiciliaria de Salud* OR *Servicios de Atención de Salud a Domicílio*) AND (*Respiración Artificial*) AND (*Terapia por Inhalación de Oxígeno*) AND
Web of Science	(Disabled children) AND (Chronic disease) AND (Home nursing OR Home Care Services) AND (Respiration, artificial) AND (Oxygen Inhalation Therapy) AND
Embase	(Disabled children) AND (Chronic disease) AND (Home nursing OR Home Care Services) AND (Respiration, artificial) AND (Oxygen Inhalation Therapy) AND
SciELO	(*Crianças com deficiência*) AND (*Doença crônica*) AND (*Assistência domiciliar* OR *Serviços de cuidado domiciliar*) AND (*Ventilação mecânica*) AND (*Oxigenoterapia*) AND (*Cuidadores*) AND

#### Stage 3 - Study selection

Eligibility criteria included original studies in English, Portuguese, and Spanish that fit the PCC model to answer the research question. The research outcome includes daily home care of children with CCCs dependent on technology.

#### Inclusion criteria

Original research, reviews, experience reports, theoretical essays, and full-text publications from the last ten years were included, provided their themes address and answer the study question. This time limit is justified by the expansion of the Better at Home Program (In Portuguese, *Programa Melhor em Casa*) and the deepening debate surrounding the deinstitutionalization of children with chronic conditions who have undergone long-term hospital stays^(10)^.

#### Exclusion criteria

Editorials, reviews, letters, case studies, undergraduate theses, duplicate works, conference proceedings, and articles not freely available in full online were excluded.

**Figure 1:**
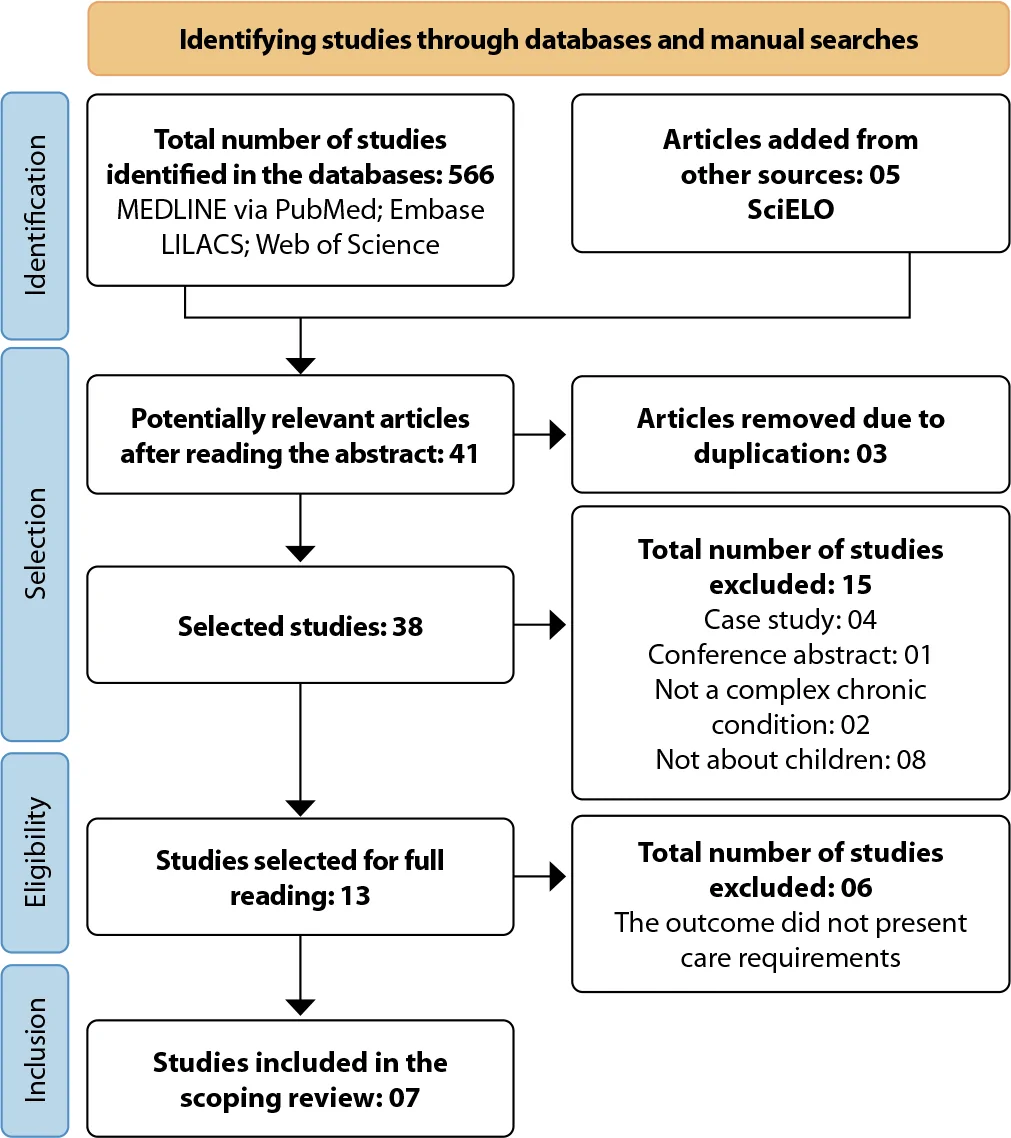
Flowchart of the Preferred Reporting Items for Systematic Reviews and Meta-Analyses extension for Scoping Reviews, Rio de Janeiro, Rio de Janeiro, Brazil, 2025

Study selection was conducted according to the PRISMA-ScR methodology^(9,11)^. To this end, a flowchart (Figure 1) was developed outlining the search and selection process for the studies in this review, showing the quantitative results for each database, studies included/excluded, and the total number of studies selected for assessment and synthesis.

The initial search identified 566 studies. After reading the titles and abstracts, 41 articles were selected according to the study question. Of these, only seven met eligibility criteria, as shown in the search and selection flowchart presented in Figure 1.

#### Stage 4 - Data mapping

Based on the data presented, we highlighted the care items by thematic area and the home care demands identified for children with CCCs dependent on technology: 1. Respiratory system care; 2. Feeding and medication administration care; 3. Ostomy care; 4. Skin care; 5. Other daily care.

#### Stage 5 - Result summary and report

The results were presented and organized in a descriptive chart (Chart 2) containing data on the authors, year, country (extracted based on information from the first author), study design, objective, outcome, and level of evidence. The quality of evidence was classified into seven levels according to Melnyk *et al.* ^(12)^. At level 1, evidence comes from systematic reviews or meta-analyses of all relevant randomized controlled clinical trials or from clinical guidelines based on systematic reviews of these trials. At level 2, evidence is derived from at least one well-designed randomized controlled clinical trial. At level 3, evidence is obtained from well-designed clinical trials, but without randomization. At level 4, evidence comes from well-designed cohort and case-control studies. At level 5, evidence originates from systematic reviews of descriptive and qualitative studies. At level 6, evidence is derived from a single descriptive or qualitative study. At level 7, evidence comes from expert opinion and/or reports from expert committees.

**Chart 2:** Description of the studies included in the review, Rio de Janeiro, Rio de Janeiro, Brazil, 2025

	Authors	Title	Year Country	Study design	Objective	Outcomes	Level of evidence
1	Xiao *et al.* ^(13)^	Long-term mechanical ventilation and transitions in care: A narrative review	2023 United States of America	Systematic review	Describe three transitions: institution/hospital to community/home; pediatric care to adult care; and active end-of-life care for ventilator-assisted individuals.	Equipment handling, basic life support, warning signs, tracheostomy tube replacement, tracheostomy suctioning, equipment cleaning, oxygen therapy, aerosol therapy, troubleshooting ventilator malfunctions, feeding, skin care.	V
2	Elias *et al.* ^(14)^	Home care of children and youth with complex health care needs and technology dependencies	2022 (2017) (2012)^*^ United States of America ^*^This clinical report was reaffirmed in May 2017 and December 2022	Clinical report	Present an approach for discharging a child with complex medical needs and technology dependencies from the hospital to home, and then continuously meeting the needs of children and families in home environments.	Enteral feeding tube management, tracheostomy care, respiratory support device (nebulizers, ventilators, etc.) and monitor management, wound care, intravenous line care, medication management, ability to assess children’s clinical conditions such as signs of worsening, aspiration of secretions in tracheostomy, adoption of measures to prevent pressure and decubitus injuries, pain assessment, and colostomy care.	VII
3	Ribeiro *et al.* ^(15)^	Home care for children with gastrostomy	2022 Brazil	Qualitative research	Describe the home care practices performed by family members to maintain the life of a child with a gastrostomy.	Food and medication administration via gastrostomy tube, tube and ostium care and management, bathing care, and hand hygiene.	VI
4	Page *et al.* ^(16)^	Training and support for caring for a child’s gastrostomy: a survey with family carers	2021 United States of America	Mixed-methods (quantitative and qualitative)	Explore the experiences of family caregivers regarding training and ongoing support for caring for their children’s gastrostomy.	Button replacement and ostium care, gastrostomy tube replacement, enteral feeding administration via gastrostomy, and medication administration via gastrostomy.	VI
5	Sobotka *et al.* ^(17)^	Discharge Practices for Children with Home Mechanical Ventilation across the United States	2020 United States of America	Cohort study	Assess discharge practices for children on invasive mechanical ventilation at home in the United States of America.	Tracheostomy exchange.	IV
6	Sterni *et al.* ^(18)^	An official ATS clinical practice guideline: pediatric chronic home invasive ventilation	2016 United States of America	Expert panel and consensus analysis	Develop evidence-based clinical practice guidelines for hospital discharge and home/community management of children requiring chronic invasive ventilation.	Obtaining basic vital signs, pulmonary care and assessment, tracheostomy care, emergency response, Ambu bag management, ventilator management, infection control practices, medication management and storage and administration care, understanding equipment alarm signals (ventilators, monitors, and infusion pumps), and enteral tube feeding and gastrostomy care.	VII
7	Aroonwan Preutthipan^(19)^	Home Mechanical Ventilation in Children	2015 India	Review	Review the literature on home mechanical ventilation in children.	Ventilator and accessory use, cleaning and care, basic problem-solving skills, emergency troubleshooting, understanding alarms and corrective measures to be taken; tracheostomy care, basic life support, suctioning through a tracheostomy or by using manual and/or mechanical cough assistance techniques.	V

## RESULTS

Of the seven studies selected, the majority (n=5) came from the United States of America, with the remainder from Brazil (n=1) and India (n=1).

The study designs included literature reviews (n=2), clinical report (n=1), cohort study (n=1), mixed-methods study (n=1), expert consensus (n=1), and qualitative research (n=1). The levels of evidence for the studies ranged from IV to VII, as can be seen in Chart 2.

The selected studies highlighted the following main outcomes: airway management, mechanical ventilation and other life support equipment; ostomy care (especially tracheostomy and gastrostomy); diet and medication administration; equipment cleaning; identification of basic and warning vital signs (clinical instability and alarms); basic life support; and wound care and postural changes. Moreover, routine daily care, such as bathing and personal hygiene, was considered (Chart 2).

## DISCUSSION

There are recommendations for the safe discharge of people with chronic conditions from the hospital, already established in publications from the Ministry of Health, regarding home care, which aim to ensure better outcomes in the transition from hospital to home. Among the recommendations, family caregiver training stands out, reporting that home caregivers should receive adequate training on specific care, such as medication administration, feeding, hygiene, vital sign monitoring, recognition of emergency signs, etc.^(6,10,20)^.

The safe care described in the Practical Guide for Caregivers^(6)^ includes feeding tube management, ostomy care, identification of emergency situations, skin lesion care, changes in position, in addition to routine care such as feeding, hygiene, among others. However, the aforementioned guide does not provide information on life support technology management and care, such as oxygen and mechanical ventilation. Family caregivers play a fundamental role in home care; therefore, they need to know and be trained, especially by the nursing staff, while still in the hospital, to continue this care for all children’s needs^(6,10,20)^.

This literature review aimed to describe the main daily home care needs of children with CCCs and dependent on technology after the hospital discharge process.

Based on a literature review, it is understood that home care for children with CCCs dependent on technology includes everything from hygiene care, positioning in bed, wound prevention, and administration of food and medication to more complex care, such as ostomy management and cleaning, mechanical ventilation, and other life support devices^(13-19)^, in accordance with Ministry of Health recommendations^(6,10)^. However, some research suggests that cardiopulmonary resuscitation training is necessary for emergencies that may occur at home^(13,14,17,18)^. Furthermore, research has described the need for at least two caregivers to be trained in home care^(13,16-18)^.

The work of Preutthipan^(19)^ and Xiao *et al.* ^(13)^ strongly believes that parents and family members are capable of caring for their children at home if they are trained, leading to a safe transition between the hospital and home. Ribeiro *et al.* ^(15)^ emphasize that parents have doubts about daily management of children with ostomies, such as bathing and hygiene. Therefore, it is suggested that this type of care should also be trained for home care during hospitalization^(15)^. As can be seen, the needs are diverse. Therefore, in order to facilitate understanding of home care, these have been grouped into major care blocks.

### Respiratory system care

Respiratory problems are among the most common complications in children with CCCs, especially in conditions such as neuromuscular diseases, complex heart conditions, pulmonary dysplasia, and genetic disorders that compromise lung function. These children may require continuous oxygen therapy, invasive or non-invasive ventilatory support, airway suctioning, and constant respiratory pattern and peripheral oxygenation monitoring^(21)^. Seddon & Khan^(22)^ highlight that children with neuromuscular disabilities, such as muscular dystrophy, may require continuous positive airway pressure ventilation, bilevel positive airway pressure or tracheostomy ventilation. Piper^(23)^ points out that oxygen supplementation is frequently indicated for children with neurological problems, as well as for those with complex congenital heart disease. Other interventions, such as regular lung toilet - often with high-frequency chest wall oscillation (e.g., cough assist) - and other devices, may be necessary, according to Piper^(23)^. Home respiratory care therefore requires technical training for caregivers, especially in device management such as tracheostomies, home mechanical ventilators, and oxygen concentrators. Furthermore, the ability to recognize early signs of respiratory distress, thick secretions, hypoxia, and airway obstruction is fundamental to preventing complications that can rapidly progress to severe conditions^(24,25)^. Rangel^(26)^ points out that continuous training of family members and home care team’s performance are determining factors for maintaining the clinical stability of these children.

Five of the seven studies in this report indicate the need for daily respiratory system care, including cleaning and management with invasive or non-invasive mechanical ventilation, oxygen therapy, airway suctioning and tracheostomies, recognition of respiratory alarms, and cough assistance^(13,14,17-19)^.

Sobotka *et al.* ^(17)^, in a cohort study, assessed the practice of hospital discharge for children on mechanical ventilation in the United States and observed the need for cannula exchange training, as this is necessary care at home. This type of care was also reported in the work of Xiao *et al.* ^(13)^ and Preutthipan^(19)^. In children with technology-dependent CCCs, changing tracheostomy tubes is fundamental to ensuring airway maintenance, preventing complications, and promoting a higher quality of life for children in home environments. However, it is a procedure that requires careful training, continuous monitoring, and support from the healthcare team. According to Lawrence *et al.* ^(27)^, empowering parents in home tracheostomy care contributes to a reduction in hospitalizations, promoting greater family autonomy in comprehensive child care. However, if scheduling with the presence of a medical professional, physiotherapist, or nurse is possible, there will be greater comfort and less risk of complications for children, such as false passage of the new tube during insertion.

Preutthipan^(19)^ describes, in a more general way, that there is a need for care with mechanical ventilators and accessories, in addition to tracheostomy cannula aspiration and the use of manual or mechanical cough assistance techniques.

Sterni *et al.* ^(18)^ created a panel of experts to develop evidence-based clinical practice guidelines for hospital discharge and home/community management of children requiring chronic invasive ventilation. The study demonstrated that home care with ventilators and the use of a manual resuscitator (Ambu) in the home respiratory care of tracheostomized children are essential strategies for managing emergency situations, such as cannula obstructions or apnea episodes.

Savassi *et al.* ^(28)^ highlight that parents, after training by the health team on how to act in emergency situations, are trained to use the Ambu bag safely, ensuring adequate ventilation until medical support arrives, when necessary. This skill is part of expanded care, which aims to avoid frequent hospitalizations and promote greater clinical stability at home. According to Pereira *et al.* ^(29)^, training caregivers in Ambu bag use is directly related to the effectiveness of home care and the safety of children in the face of respiratory problems.

### Food and medication administration care

Nutrition in children with CCCs is often compromised by factors such as dysphagia, gastroesophageal reflux, difficulties with sucking and swallowing, and dependence on enteral nutrition via nasogastric tube or gastrostomy. Maintaining adequate nutritional status requires not only prescribing a diet compatible with children’s clinical needs, but also caregivers mastering correct administration techniques, device hygiene, and prevention of complications such as infections, vomiting, and aspiration. When oral intake is contraindicated, it can be administered via gastrostomy tube, nasogastric tube, nasojejunal tube, or jejunal tube^(30)^. Medications can also be administered through these routes.

Continuous medication use, often in complex regimens, demands rigorous organization of home routines, with extra attention to schedules, dosages, drug interactions, and adverse reactions^(31)^. Children with special care needs receive five times more medication than typical children, and medication management is placed as a responsibility of family care at home by Elias *et al.* ^(14)^.

Xiao *et al.* ^(13)^ point to aerosol therapy as a care measure. Sterni *et al.* ^(18)^ discuss medication management and care with its storage and administration. Elias *et al.* ^(14)^ point out that the enteral feeding tube management is a home care measure that may be necessary for children with CCCs.

### Ostomy care

Ostomies - such as tracheostomies, gastrostomies, and colostomies - represent a central aspect of home care for children with CCCs dependent on technology, and are frequently associated with respiratory, nutritional, and digestive needs. Proper management of these devices requires technical knowledge of peristomal skin care, device exchange and maintenance, infection prevention, and recognition of signs of complications such as strictures, prolapses, or extrusions^(32)^.

Gastrostomy is a surgical procedure that creates an opening in the abdomen to connect the stomach to the skin. Gastrostomy feeding can be administered by gravity (gavage) or via an infusion pump. Care should be taken to clean the gavage syringe and the stoma after bathing, and to dry the area to avoid excessive moisture, as this can promote skin irritation and fungal infection^(33)^. A colostomy is a surgical opening made in the colon, externalized on the abdominal wall, allowing for the elimination of feces through a collection bag. This procedure can be temporary or permanent, and is indicated in cases of anorectal malformations, inflammatory bowel diseases, obstructions, or trauma, and requires specific care to prevent infections^(34,35)^.

According to Ricz *et al.* ^(36)^, tracheostomy is a surgical procedure that involves opening the trachea to the skin in order to ensure an airway, and its opening is supported by a tracheostomy cannula, which facilitates breathing and secretion removal. In a study by Elias *et al.* ^(15)^, it is recommended to monitor the tracheostomy inflation pressure daily to avoid lesions in the tracheal epithelium, in addition to cleaning the cannula. All these care measures should be performed by caregivers at home.

Page *et al.* ^(16)^ and Ribeiro *et al.* ^(15)^ explored experiences in gastrostomy care management. These studies pointed out that there is a need for home care regarding button changes, gastrostomy care, administration of food and medication, and even bathing care. Elias *et al.* ^(14)^ add colostomy care.

Ribeiro *et al.* ^(15)^ also point to the need for careful hand hygiene before handling this device. Hand hygiene is an essential measure in preventing infections and ensuring the safety of children with CCCs who require respiratory care and feeding devices at home. Proper handling of this equipment requires rigorous hygiene practices to minimize the risk of contamination and respiratory complications^(37,38)^.

### Skin care related care

Skin integrity is a critical aspect of home care for technology-dependent children with CCCs, especially due to the presence of invasive medical devices, prolonged immobility, and the constant use of diapers or dressings. Pressure sores, irritative dermatitis, fungal infections, and ulcers are frequent complications that negatively impact quality of life and increase the risk of hospitalizations^(39)^.

Preventing these complications requires multidimensional strategies, including proper hygiene, skin hydration, frequent changes in position, use of supportive surfaces, and constant monitoring for hyperemic areas or loss of skin integrity. In this context, the nursing team plays an essential role in educating caregivers and implementing preventive care protocols^(40)^.

Elias *et al.* ^(14)^, in their clinical report, show that children with mobility limitations are vulnerable to the appearance of pressure injuries and compromised skin integrity. Therefore, among the daily care, there is a need for regular changes of position and protection of bony prominences, particularly in the posterior occipital region, sacrum, ischium and heels.

According to recommendations from the Brazilian Ministry of Health, the clinical management of research by Elias *et al.* ^(14)^ places the care of changing decubitus and preventing skin injury as an attribution of caregivers of children with CCCs dependent on technology.

### Other daily home care

Other care considerations that appear in this literature search relate to vital sign monitoring, cited by Xiao *et al.* ^(13)^ and Sterni *et al.* ^(18)^, as well as the identification of warning signs. Continuous clinical parameter monitoring constitutes an essential dimension of home care for children with CCCs, since it allows for the early detection of clinical changes and timely intervention in the event of complications. Vital sign monitoring, such as body temperature, heart rate, respiratory rate, and oxygen saturation, should be carried out systematically and documented by caregivers, with the support and guidance of the health team. Devices such as pulse oximeters, digital thermometers, and portable monitors have been frequently used in this context^(41)^.

Xiao *et al.* ^(13)^ and Elias *et al.* ^(14)^ highlight the importance of identifying signs of aggravation or pain, emergency care, and problem-solving. Identifying warning signs, such as sudden changes in level of consciousness, cyanosis, persistent fever, seizures, intractable vomiting, respiratory distress, among others, requires constant attention and adequate training of family members. The ability to recognize these signs and seek timely care is a decisive factor in preventing complications and hospitalizations^(42)^.

Another relevant aspect concerns pain assessment and management, since many children with CCCs live with chronic pain associated with procedures, sustained postures, spasticity, or underlying diseases. Pain assessment should be performed using scales appropriate to children’s age and neuropsychomotor development, such as the Face, Legs, Activity, Cry, Consolability scale for nonverbal children. Appropriate pain management includes both pharmacological interventions and non-pharmacological measures, such as massage, proper positioning, and environmental comfort^(42,43)^.

Furthermore, it is essential that caregivers are trained to act in urgent and emergency situations, such as airway suctioning, device disconnection, respiratory arrest, or catheter obstruction. Action protocols, checklists, and first aid training are strategies that should be incorporated into the home routine, promoting greater safety and readiness to respond^(25)^.

Finally, Ribeiro *et al.* ^(16)^ point out in their study that daily care, such as personal hygiene, changing clothes, maintaining a sleep routine, stimulating neuropsychomotor development, and attending to children’s emotional well-being, make up the daily routine of home care. These often-invisible care practices are extremely important to guarantee dignity, comfort, and quality of life for the child and the family. Therefore, recognizing the complexity of daily care and providing systematic support to families should be premises of home care services^(44-47)^.

### Study limitations

The study had limitations related to the scarcity of published literature on the subject in the last ten years.

### Contributions to health, nursing, or public policy

This study presents a relevant and current contribution to the field of pediatric nursing and home care, and introduces the premises of personand family-centered care practice from the perspective of child health, which recognizes the family as central to children’s lives, views children within the context of their family, and supports family members in their role as caregivers. The description of daily care at home for children with CCCs, combined with the partnership between healthcare providers (such as health professionals and family members), can contribute to the development of a comprehensive and personalized care plan, as well as to decision-making and caregiver training. This provides nurses with the necessary content for structuring training programs, creating instructional materials (such as booklets and care checklists), and disseminating best practices for the care of children with CCCs at home.

It is important to support family caregivers in providing safe and harm-free care, aimed at maintaining the life, development, and comfort of their children.

## CONCLUSIONS

The review of published studies made it possible to identify various daily care needs for children with CCCs dependent on technology at different levels of complexity. Family caregiver preparation takes place in a hospital setting permeated by problem-solving educational practices, based on dialogue and the execution of essential activities performed and practiced on the spot, where nurses need to negotiate knowledge that is not part of the daily care of these families. Therefore, nurses must be the coordinator of the educational process, actively participating in preparing the family to care for children with CCCs at home.

The complexity of home care demands for children with CCCs requires not only technical procedural skills but also a family-centered approach to daily life routines that recognizes the specificities of the home context to promote caregiver autonomy and empowerment. Respiratory system care, food and medication administration, ostomy care, and skin integrity care constitutes critical areas requiring continuous support, continuing education, and coordination between different levels of healthcare. Nursing care assessment is based on the integration of health promotion with socio-environmental, cultural, and personal factors that affect the well-being of the child or adolescent and their family.

Taking responsibility for the care and management of technology-dependent children is a life-altering event for most families and caregivers. However, training for parents and family members as caregivers was reported in all studies in this review as very important and relevant for successful home-based experiences. This information may assist in proposing a training program for parents who are caregivers of technology-dependent children requiring long hospital stays.

## Data Availability

The research data are available within the article.
