# Supplementary material for: Daily care demands of children with complex chronic conditions at home: a scoping review
Source: Rev Bras Enferm. 2026 Sep 18;79:e20250341. doi: 10.1590/0034-7167-2025-0341 (PMC13588627; doi:10.1590/0034-7167-2025-0341)
Supplement: Translation

# Translation: Demandas de cuidados diários de crianças com condição crônica complexa no domicílio: revisão de escopo

**DOI:** 10.1590/0034-7167-2025-0341pt

**Authors:** Tânia Barroso Barreto, Viviane Medeiros Machado, Mariana Setúbal Nassar de Carvalho, Carla Trevisan Martins Ribeiro

**Affiliations:** I Instituto Nacional da Saúde da Mulher da Criança e do Adolescente Fernandes Figueira/FIOCRUZ. Rio de Janeiro, Rio de Janeiro, Brasil

**Keywords:** Crianças com Deficiência, Doença Crônica, Assistência Domiciliar, Ventilação Mecânica, Cuidadores.

## Abstract

**Objetivos:**

descrever as principais demandas de cuidados domiciliares de crianças com condições crônicas complexas e dependentes de tecnologia após a desospitalização.

**Métodos:**

revisão de escopo com foco nos cuidados necessários no domicílio para crianças com condições crônicas complexas com dependência tecnológica. A busca foi realizada utilizando os termos “criança com deficiência”, “doença crônica”, “assistência domiciliar”, “ventilação mecânica” e “cuidadores”, além de similares em inglês e espanhol, considerando os últimos dez anos.

**Resultados:**

incluíram-se sete estudos. Identificaram-se como principais cuidados manejo das vias aéreas, ventilação mecânica, equipamentos de suporte à vida, ostomias, administração de dieta e medicamentos, higienização de equipamentos, monitoramento de sinais vitais, suporte básico de vida, cuidados com feridas, mudanças posturais e higiene.

**Conclusões:**

as demandas de crianças com condições crônicas complexas no domicílio exigem conhecimento técnico e adaptação das rotinas singulares. Essas informações contribuem para o desenvolvimento de programas de treinamento para cuidadores, tendo o enfermeiro como articulador.

## INTRODUÇÃO

O perfil epidemiológico das crianças hospitalizadas no Brasil e no mundo se transformou nas últimas décadas, com o aumento expressivo de internações prolongadas, especialmente devido à maior sobrevida de crianças com condição crônica de saúde e à dependência de tecnologias por longo período de tempo^(1)^. Entretanto, a longa permanência dessas crianças nos leitos hospitalares traz fortes impactos na falta de leitos em Unidades de Terapia Intensiva, alto custo da assistência, além de consequências sobre o desenvolvimento da criança e a manutenção da unidade familiar, o que leva à necessidade eminente de desospitalização desta população^(2)^.

A condição crônica complexa (CCC) pode ser definida como doenças que possuem sequelas permanentes e que necessitam de cuidados por longos períodos, muitas vezes durante todo o ciclo vital do indivíduo, em um modelo que deve ter acesso a tecnologias de suporte de vida, como ventilação não invasiva, dispositivos para traqueostomias e gastrostomias, entre outros^(3)^. Entende-se, dessa forma, que doença crônica é uma condição de saúde com repercussões em diferentes estruturas e funções corporais, que habitualmente não é fatal, mas que demanda cuidados múltiplos e complexos^(2)^.

No Brasil, os programas e serviços hospitalares pediátricos de referência para atenção integral às crianças com CCCs alicerçam sua principal estratégia organizacional na coordenação do cuidado integral visando à desospitalização, pois comprovadamente tem os melhores desfechos na saúde e no bem-estar delas e de suas famílias^(1)^. A desospitalização dessa população, muitas vezes, é um desafio para as equipes de saúde, sendo essencial uma alta planejada e coordenada, com mapeamento da rede de apoio e da rede de assistência próxima à residência, bem como treinamento para os cuidados diários ainda no período da hospitalização. É importante que os cuidadores aprendam sobre os cuidados diários com as crianças, desde o correto posicionamento no leito/berço ou a importância da lavagem de mãos até procedimentos como aspiração, gavagem de dietas e troca de curativos, pois serão os responsáveis por estes cuidados no domicílio^(4)^.

O treinamento do cuidador familiar vem se tornando uma grande ferramenta para desospitalização segura de crianças e adolescentes com CCCs. Mesmo com o acompanhamento de equipe multiprofissional com visitas frequentes no domicílio após a alta orientada e pactuada com o território, a família ocupa papel importante no protagonismo do cuidado diário^(5)^. Uma vez que os cuidadores familiares precisam de um preparo durante o processo de desospitalização, para que possam prestar os cuidados necessários às crianças no domicílio com segurança, assumindo o protagonismo deste cuidado, uma questão vem à tona: quais as demandas de cuidados no domicílio as crianças com CCCs necessitam*?*

O Ministério da Saúde publicou em 2008 um Guia Prático do Cuidador^(6)^ que tem como objetivo orientar cuidadores na atenção à saúde das pessoas de qualquer idade, acamadas ou com limitações físicas, que necessitam de cuidados especiais para o cuidado no domicílio. Contudo, este guia, além de antigo, não é destinado especificamente à população infantil. Torna-se então imprescindível uma busca de pesquisas e *guidelines* internacionais que tragam informações acerca do objetivo do estudo: necessidades de cuidados domiciliares de crianças com CCCs dependentes de tecnologia.

## OBJETIVOS

Descrever as principais demandas de cuidados diários domiciliares de crianças com CCCs e dependentes de tecnologia após o processo de desospitalização.

## MÉTODOS

Trata-se de revisão de escopo, realizada de outubro de 2024 a fevereiro de 2025, e conduzida com base nas diretrizes do Manual de Síntese de Evidências do JBI^(7)^, seguindo cinco etapas: 1 - descrição da questão de pesquisa; 2 - identificação de estudos relevantes; 3 - seleção dos estudos; 4 - mapeamento de dados; e 5 - resumo e relato dos resultados. O protocolo para esta revisão foi registrado no PROSPERO (https://www.crd.york.ac.uk/PROSPERO/view/CRD420251044808) e no *Open Science Framework* (https://doi.org/10.17605/OSF.IO/NXRK8). A revisão foi realizada de acordo com a *Preferred Reporting Items for Systematic reviews and Meta-Analyses extension for Scoping Reviews* (PRISMA- ScR)^(7-9)^.

### Etapas da revisão de escopo:

#### Etapa 1 - Descrição da questão de pesquisa

A primeira etapa compreendeu a elaboração de uma pergunta de pesquisa de acordo com o acrônimo PCC (População (crianças com CCCs dependentes de tecnologias após processo de desospitalização), Conceito (demandas de cuidados diários) e Contexto (cuidado domiciliar por cuidadores/pais)), a saber: quais são os cuidados diários necessários no domicílio às crianças com CCCs dependentes de tecnologias após processo de desospitalização?

#### Etapa 2 - Identificação dos estudos relevantes

A segunda etapa compreendeu a escolha das bases de dados e elaboração das estratégias de busca com descritores e operadores booleanos.

Os descritores do vocabulário controlado foram selecionados a partir dos Descritores em Ciências da Saúde, *Medical Subject Headings*, CINAHL e Emtree/Embase. A busca bibliográfica foi realizada de outubro a dezembro de 2024 nas bases de dados *US National Library of Medicine National Institutes of Health* (PubMed Central), Literatura Latino-Americana e do Caribe em Ciências da Saúde (LILACS), *Web of Science*, Embase e *Scientific Electronic Library Online* (SciELO) (Quadro 1).

**Quadro 1:** Estratégia de Busca com descritores, Rio de Janeiro, Rio de Janeiro, Brasil, 2025

MEDLINE via PubMed	*(Disabled children) AND (Chronic disease) AND (Home nursing OR Home Care Services) AND (Respiration, artificial) AND (Oxygen Inhalation Therapy) AND*
LILACS	*(Niños con Discapacidad) AND (Enfermedad Crónica) AND (Atención Domiciliaria de Salud OR* *Servicios de Atención de Salud a Domicílio) AND (Respiración Artificial) AND* *(Terapia por Inhalación de Oxígeno) AND*
*Web of Science*	*(Disabled children) AND (Chronic disease) AND (Home nursing OR Home Care Services) AND (Respiration, artificial) AND (Oxygen Inhalation Therapy) AND*
Embase	*(Disabled children) AND (Chronic disease) AND (Home nursing OR Home Care Services) AND (Respiration, artificial) AND (Oxygen Inhalation Therapy) AND*
SciELO	(Crianças com deficiência) *AND* (Doença crônica) *AND* (Assistência domiciliar *OR* Serviços de cuidado domiciliar) *AND* (Ventilação mecânica) *AND* (Oxigenoterapia) *AND* (Cuidadores) *AND*

#### Etapa 3 - Seleção dos estudos

Os critérios de elegibilidade incluíram estudos originais em inglês, português e espanhol, que se encaixassem no modelo PCC, para responder à pergunta da pesquisa. O desfecho da pesquisa inclui cuidados domiciliares diários de crianças com CCCs dependentes de tecnologia.

#### Critérios de inclusão

Incluíram-se pesquisas originais, revisões, relatos de experiências, ensaios teóricos, publicações na íntegra, no período dos últimos dez anos, cuja temática aborde e responda à questão do estudo. Justifica-se esse limite temporal pela expansão do Programa Melhor em Casa e pelo aprofundamento do debate em torno da desospitalização de crianças com condições crônicas em internação hospitalar de longa permanência^(10)^.

#### Critérios de exclusão

Excluíram-se editoriais, resenhas, cartas, estudos de caso, trabalhos de conclusão de curso de graduação, aqueles trabalhos em duplicidade, anais de congresso e artigos não disponíveis gratuitamente e *online* na íntegra.

A seleção dos estudos foi conduzida conforme a metodologia PRISMA-ScR^(9,11)^. Para isso, foi elaborado um fluxograma (Figura 1) sobre o processo de busca e seleção dos estudos desta revisão, com os resultados quantitativos de cada base de dados, estudos incluídos/excluídos e o total de trabalhos selecionados para avaliação e síntese.

**Figura 1:** Diagrama de fluxo do *Preferred Reporting Items for Systematic reviews and Meta-Analyses extension for Scoping Reviews*, Rio de Janeiro, Rio de Janeiro, Brasil, 2025

A busca inicial identificou 566 estudos. Após a leitura dos títulos e resumos, foram selecionados 41 artigos de acordo com a pergunta do estudo. Destes, apenas sete preencheram os critérios de elegibilidade, conforme o fluxograma de busca e seleção apresentado na Figura 1.

#### Etapa 4 - Mapeamento dos dados

Com base nos dados apresentados, destacamos os itens de cuidados por área temática e as demandas de cuidados domiciliares levantadas para crianças com CCCs dependentes de tecnologias: 1. Cuidados relacionados ao sistema respiratório; 2. Cuidados relacionados à administração de alimentação e medicação; 3. Cuidados com ostomias; 4. Cuidados com a pele; 5. Outros cuidados diários.

#### Etapa 5 - Resumo e relato dos resultados

Os resultados foram apresentados e organizados em quadro descritivo (Quadro 2) contendo dados dos autores, ano, país (extraído com base nas informações do primeiro autor), tipo de estudo, objetivo, desfecho e nível de evidência. A qualidade das evidências foi classificada em sete níveis segundo Melnyk *et al*.^(12)^. No nível 1, as evidências são provenientes de revisão sistemática ou metanálise de todos os ensaios clínicos randomizados controlados relevantes ou oriundas de diretrizes clínicas baseadas em revisões sistemáticas desses ensaios. No nível 2, as evidências são derivadas de pelo menos um ensaio clínico randomizado controlado bem delineado. No nível 3, as evidências são obtidas de ensaios clínicos bem delineados, mas sem randomização. No nível 4, as evidências são provenientes de estudos de coorte e de caso-controle bem delineados. No nível 5, as evidências são originárias de revisão sistemática de estudos descritivos e qualitativos. No nível 6, as evidências são derivadas de um único estudo descritivo ou qualitativo. No nível 7, as evidências são oriundas de opinião de autoridades e/ou relatórios de comitês de especialistas.

**Quadro 2:** Descrição dos estudos incluídos na revisão, Rio de Janeiro, Rio de Janeiro, Brasil, 2025

	Autores	Título	Ano País	Tipo de estudo	Objetivo	Desfechos	Nível de evidência
1	Xiao *et al*.^(13)^	*Long-term mechanical ventilation and transitions in care: A narrative review*	2023 Estados Unidos da América	Revisão sistemática	Descrever três transições: instituição/hospital para comunidade/lar; cuidado pediátrico para cuidado adulto; e tratamento ativo para fim de vida para indivíduos assistidos por ventilador.	Manejo de equipamentos, suporte básico de vida, sinais de alerta, substituição da cânula de traqueostomia, aspiração de traqueostomia, limpeza dos equipamentos, oxigenoterapia, terapia por aerossol, resolução de problemas de mau funcionamento do ventilador, alimentação, cuidados com a pele.	V
2	Elias *et al*.^(14)^	*Home care of children and youth with complex health care needs and technology dependencies*	2022 (2017) (2012)^*^ Estados Unidos da América ^*^Este relatório clínico foi reafirmado em maio de 2017 e dezembro de 2022	Relatório clínico	Apresentar uma abordagem para dar alta à criança com necessidades médicas complexas com dependências de tecnologia do hospital para casa e, em seguida, atender continuamente às necessidades da criança e da família no ambiente doméstico.	Manejo de tubos de alimentação enteral, cuidados com traqueostomia, manejo de suportes respiratórios (nebulizadores, ventiladores, *etc*.) e monitores, cuidados com feridas, cuidados com linha intravenosa, gerenciamento de medicamentos, saber avaliar as condições clínicas da criança como sinais de agravo, aspiração da secreção em traqueostomia, adoção de medidas de prevenção de lesões de pressão e de decúbito, avaliação da dor, e cuidados com colostomias.	VII
3	Ribeiro *et al*.^(15)^	*Home care for children with gastrostomy*	2022 Brasil	Pesquisa qualitativa	Descrever as práticas de cuidados domiciliares realizados por familiares para manutenção da vida de criança com gastrostomia.	Administração de alimentação e medicação pela sonda de gastrostomia, cuidados e manejo com a sonda e óstio, cuidados com o banho, e higienização das mãos.	VI
4	Page *et al*.^(16)^	*Training and support for caring for a child’s gastrostomy: a survey with family carers*	2021 Estados Unidos da América	Métodos mistos, (quantitativo e qualitativo)	Explorar as experiências de cuidadores familiares em relação ao treinamento e suporte contínuo para cuidar da gastrostomia de seus filhos.	Troca de boton e cuidados com óstio, troca de sonda de gastrostomia, administração da alimentação enteral por gastrostomia, e administração de medicamentos por gastrostomia.	VI
5	Sobotka *et al*.^(17)^	*Discharge Practices for Children with Home Mechanical Ventilation across the United States*	2020 Estados Unidos da América	Estudo de coorte	Avaliar a prática de alta para crianças com ventilação mecânica invasiva domiciliar nos Estados Unidos da América.	Troca de traqueostomia.	IV
6	Sterni *et al*.^(18)^	*An official ATS clinical practice guideline: pediatric chronic home invasive ventilation*	2016 Estados Unidos da América	Painel de especialistas e análise de consenso	Desenvolver diretrizes de prática clínica baseadas em evidências para a alta hospitalar e o manejo domiciliar/comunitário de crianças que necessitam de ventilação invasiva crônica.	Obtenção de sinais vitais básicos, cuidados e avaliação pulmonar, cuidados com traqueostomia, respostas a emergências, manejo do Ambu, manejo do ventilador, práticas de controle de infecção, manejo de medicamentos e cuidados com a conservação e administração, compreensão dos sinais de alarme dos equipamentos (ventiladores e monitores e bombas infusoras), e cuidados com alimentação por sonda enteral e gastrostomia.	VII
7	Aroonwan Preutthipan^(19)^	*Home Mechanical Ventilation in Children*	2015 Índia	Revisão	Revisar a literatura sobre ventilação mecânica domiciliar em crianças.	Uso, limpeza e cuidados com os ventiladores e acessórios, habilidades básicas de resolução de problemas, resolução de problemas de emergência, compreensão de alarmes e medidas corretivas a serem tomadas; cuidados com traqueostomia, suporte básico de vida, aspiração através de uma traqueostomia ou pelo uso de técnicas manuais e/ou mecânicas de assistência à tosse.	V

## RESULTADOS

Dos sete estudos selecionados, a maioria (n= 05) era proveniente dos Estados Unidos da América, sendo o restante do Brasil (n=1) e da Índia (n=1).

Os desenhos dos estudos incluíram revisões de literatura (n=2), relatório clínico (n=1), estudo de coorte (n=1), estudo misto (n=1), consenso de especialistas (n=1), pesquisa qualitativa (n=1). Os níveis de evidências dos estudos variaram de IV a VII, como pode ser observado no Quadro 2.

Os estudos selecionados apontaram os seguintes desfechos principais: cuidados com as vias aéreas, ventilação mecânica e demais equipamentos de suporte à vida; cuidados com ostomias (especialmente traqueostomia e gastrostomia); administração de dieta e medicação; limpeza com equipamento; identificação de sinais vitais básicos e de alerta (instabilidade clínica e alarmes); suporte básico de vida; e cuidados com feridas e trocas posturais. Além disso, foram considerados os cuidados cotidianos e habituais, como banho e higiene pessoal (Quadro 2).

## DISCUSSÃO

Existem recomendações para a desospitalização segura de pessoas com condições crônicas, já determinadas nas publicações do Ministério da Saúde, no que diz respeito ao cuidado domiciliar, que visam garantir melhores desfechos na transição do ambiente hospitalar para o domicílio. Entre as recomendações, destaca-se o treinamento do cuidador familiar, que reporta que os cuidadores domiciliares devem receber treinamento adequado sobre cuidados específicos, como administração de medicamentos, alimentação, higiene, aferição de sinais vitais, reconhecimento de sinais de emergência, *etc*.^(6,10,20)^.

O cuidado seguro descrito no Guia Prático do Cuidador^(6)^ compreende manejo de sondas para alimentação, cuidado com ostomias, identificação de situações de emergência, cuidados com lesões de pele, mudanças de decúbito além dos cuidados rotineiros habituais como alimentação, higiene, entre outros. Contudo, o referido guia não traz informações sobre manejo e cuidados com tecnologias de suporte de vida, como oxigênio e ventilação mecânica. Os cuidadores familiares têm atuação fundamental no cuidado domiciliar; para tanto, precisam conhecer e serem treinados, especialmente pela equipe de enfermagem, ainda no hospital, para dar continuidade a esses cuidados em todas as necessidades da criança^(6,10,20)^.

Com esta revisão de literatura, buscou-se descrever as principais demandas de cuidados diários domiciliares de crianças com CCCs e dependentes de tecnologia após o processo de desospitalização.

A partir da revisão da literatura, compreende-se que os cuidados domiciliares de crianças com CCCs dependentes de tecnologia incluem desde cuidados de higiene, posicionamento no leito, prevenção de feridas e administração de alimentação e medicamentos até cuidados mais complexos, como manejo e limpeza de ostomias, ventilação mecânica e demais aparelhos de suporte à vida^(13-19)^, estando de acordo com as recomendações do Ministério da Saúde^(6,10)^. Contudo, algumas pesquisas apontam que há necessidade de treinamento de ressuscitação cardiorespiratória para eventualidades que ocorram no domicílio^(13,14,17,18)^. Além disso, as pesquisas descreveram a necessidade de pelo menos dois cuidadores serem treinados para os cuidados domiciliares^(13,16-18)^.

Os trabalhos de Preutthipan^(19)^ e Xiao *et al*.^(13)^ acreditam firmemente que pais e familiares são capazes de cuidar de seus filhos em casa se forem treinados, levando a uma transição segura entre o hospital e o domicílio. Ribeiro *et al*.^(15)^ ressaltam que os pais têm dúvidas sobre manejos cotidianos de crianças com ostomias, como banho e higiene. Dessa forma, sugere-se que este tipo de cuidado também seja treinado para o domicílio durante a internação^(15)^. Como pode ser observado, são diversas as necessidades. Desse modo, a fim de facilitar a compreensão sobre os cuidados domiciliares, estes foram agrupados em grandes blocos de cuidados.

## Cuidados relacionados ao sistema respiratório

As alterações respiratórias estão entre as complicações mais comuns em crianças com CCCs, especialmente em quadros como doenças neuromusculares, cardiopatias complexas, displasias pulmonares e distúrbios genéticos, que comprometem a função pulmonar. Tais crianças podem necessitar de oxigenoterapia contínua, suporte ventilatório invasivo ou não invasivo, aspiração de vias aéreas, além de monitoramento constante dos padrões respiratórios e da oxigenação periférica^(21)^. Seddon & Khan^(22)^ destacam que as crianças com deficiências neuromusculares, como distrofia muscular, podem necessitar de assistência ventilatória por pressão positiva contínua nas vias aéreas, por pressão positiva em dois níveis nas vias aéreas ou ventilados em traqueostomia. Piper^(23)^ aponta que a suplementação de oxigênio é frequentemente indicada para crianças com problemas neurológicos, bem como para aquelas com doença cardíaca congênita complexa. Outras intervenções, como a toalete pulmonar regular - muitas vezes com oscilação de alta frequência da parede torácica (por exemplo, *cough assist*) - e outros dispositivos, podem ser necessárias, segundo Piper^(23)^. O cuidado respiratório em domicílio requer, então, preparo técnico dos cuidadores, especialmente no manejo de dispositivos como traqueostomias, ventiladores mecânicos domiciliares e concentradores de oxigênio. Além disso, é fundamental a capacidade de reconhecer sinais precoces de desconforto respiratório, secreções espessas, hipóxia e obstruções das vias aéreas, a fim de prevenir complicações que possam evoluir rapidamente para quadros graves^(24,25)^. Rangel^(26)^ aponta que a capacitação contínua dos familiares e a atuação da equipe de atenção domiciliar são fatores determinantes para a manutenção da estabilidade clínica dessas crianças.

Cinco das sete pesquisas deste estudo apontam a necessidade de cuidados diários com o sistema respiratório, incluindo limpeza e manejo com ventilação mecânica invasiva ou não invasiva, oxigenoterapia, aspiração de vias aéreas e traqueostomias, reconhecimento de alarmes e assistência à tosse^(13,14,17-19)^.

Sobotka *et al*.^(17)^, em estudo de coorte, avaliaram a prática da alta hospitalar para crianças em ventilação mecânica no Estados Unidos e observaram a necessidade de treinamento de troca de cânula, pois é cuidado necessário no domicílio. Este tipo de cuidado também foi reportado no trabalho de Xiao *et al*.^(13)^ e Preutthipan^(19)^. A troca de cânula traqueal em crianças com CCCs dependentes de tecnologia é fundamental para garantir a manutenção da via aérea, prevenir complicações e promover maior qualidade de vida para a criança no ambiente familiar, porém é um procedimento que requer treinamento cuidadoso, acompanhamento contínuo e suporte da equipe de saúde. Segundo Lawrence *et al*.^(27)^, o empoderamento dos pais no cuidado com a traqueostomia domiciliar contribui para a redução de hospitalizações, promovendo maior autonomia da família no cuidado integral à criança. Entretanto, havendo a possibilidade de agendamento com a presença de um profissional médico, fisioterapeuta ou enfermeiro, haverá mais conforto e menor risco para a criança de intercorrências, como o falso trajeto da nova cânula ao ser introduzida.

Preutthipan^(19)^ descreve, de forma mais geral, que há necessidade de cuidados com os ventiladores mecânicos e acessórios, além de aspiração da cânula de traqueostomia e uso de técnicas manuais ou mecânicas de assistência a tosse.

Sterni *et al*.^(18)^ criaram um painel de especialistas para desenvolver diretrizes de prática clínica baseadas em evidências para a alta hospitalar e o manejo domiciliar/comunitário de crianças que necessitam de ventilação invasiva crônica. O estudo demonstrou que os cuidados domiciliares com ventiladores e o uso de um ressuscitador manual (Ambu) no atendimento respiratório domiciliar de crianças traqueostomizadas são estratégias essenciais para o manejo de situações de emergência, como obstruções da cânula ou episódios de apneia.

Savassi *et al*.^(28)^ destacam que os pais, após treinamento pela equipe de saúde de como atuar em situações de emergência, são capacitados a utilizar o Ambu de forma segura, garantindo a ventilação adequada até a chegada de suporte médico, quando necessário. Essa habilidade é parte do cuidado ampliado, que visa evitar hospitalizações frequentes e promover maior estabilidade clínica no domicílio. De acordo com Pereira *et al*.^(29)^, a capacitação dos cuidadores para o uso do Ambu está diretamente relacionada à eficácia do cuidado domiciliar e à segurança da criança diante de intercorrências respiratórias.

### Cuidados relacionados à administração de alimentação e medicação

A alimentação de crianças com CCCs, muitas vezes, está comprometida por fatores como disfagia, refluxo gastroesofágico, dificuldades de sucção e deglutição, além da dependência de nutrição enteral por sonda nasogástrica ou gastrostomia. A manutenção do estado nutricional adequado exige não apenas a prescrição de uma dieta compatível com as necessidades clínicas da criança, mas também o domínio, por parte dos cuidadores, de técnicas corretas de administração, higienização de dispositivos e prevenção de complicações, como infecções, vômitos e broncoaspiração. Quando a ingestão oral é contraindicada, ela pode ser administrada por sonda de gastrostomia, sonda nasogástrica, tubos nasojejunais ou jejunais^(30)^. Medicações também podem ser administradas nessas vias.

O uso contínuo de medicamentos, muitas vezes em regimes complexos, demanda uma organização rigorosa das rotinas domiciliares, com atenção redobrada para horários, dosagens, interações medicamentosas e reações adversas^(31)^. Crianças com necessidades especiais de cuidados recebem cinco vezes mais medicamentos que crianças típicas, e o gerenciamento de medicações é colocado como uma responsabilidade de cuidado do familiar no domicílio por Elias *et al*.^(14)^.

Xiao *et al*.^(13)^ apontam como um cuidado a terapia de aerossol. Já Sterni *et al*.^(18)^ falam do manejo de medicamentos e cuidados com sua conservação e administração. Elias *et al*.^(14)^ apontam que o manejo de tubos de alimentação enteral é um cuidado domiciliar que pode ser necessário às crianças com CCCs.

### Cuidados com ostomias

As ostomias - como traqueostomias, gastrostomias e colostomias - representam um aspecto central do cuidado domiciliar de crianças com CCCs dependentes de tecnologia, estando frequentemente associadas a necessidades respiratórias, nutricionais e digestivas. O manejo adequado desses dispositivos requer conhecimento técnico sobre cuidados com a pele periestoma, troca e manutenção de dispositivos, prevenção de infecções, além do reconhecimento de sinais de complicações, como estenoses, prolapsos ou extrusões^(32)^.

A gastrostomia é um procedimento cirúrgico que cria uma abertura no abdômen para comunicar o estômago com a pele. A alimentação por gastrostomia pode ser administrada pela ação da gravidade (gavagem) ou por meio de bomba infusora. Devem ser realizados cuidados com a limpeza da seringa da gavagem e do óstio após o banho, com a secagem do local para evitar o excesso de umidade, considerando que pode favorecer a irritação cutânea e infecção fúngica^(33)^. A colostomia é uma abertura cirúrgica realizada no cólon, exteriorizada na parede abdominal, permitindo a eliminação de fezes por meio de uma bolsa coletora. Essa intervenção pode ser temporária ou permanente, sendo indicada em casos de malformações anorretais, doenças inflamatórias intestinais, obstruções ou traumas, e necessitam de cuidados específicos para evitar infecções^(34,35)^.

Já a traqueostomia, segundo Ricz *et al.* ^(36)^, é um procedimento cirúrgico que consiste na abertura da traqueia para a pele de forma a garantir a via aérea, e a sua abertura é suportada por uma cânula de traqueostomia, que favorece a respiração e remoção de secreção. No estudo de Elias *et al.* ^(14)^, recomenda-se monitorar diariamente a pressão de insuflação da traqueostomia para evitar lesões no epitélio da traqueia, além de higienização da cânula. Todos esses cuidados devem ser realizados pelos cuidadores no domicílio.

Page *et al*.^(16)^ e Ribeiro *et al.* ^(15)^ exploraram as experiências dos cuidados no manejo da gastrostomia. Esses estudos apontaram que existe a necessidade de um cuidado domiciliar com relação à troca de botton, cuidado com a gastrostomia, administração de alimentação e medicação, e até mesmo cuidado com o banho. Já Elias *et al*.^(14)^ acrescentam o cuidado com as colostomias.

Ribeiro *et al.* ^(15)^ apontam inclusive, à necessidade de cuidado de higienização das mãos antes da manipulação desse dispositivo. A higienização das mãos é uma medida essencial na prevenção de infecções e na garantia da segurança das crianças com CCCs que necessitam de dispositivos de cuidado respiratório e de alimentação no domicílio. O adequado manuseio desses equipamentos exige práticas rigorosas de higiene para minimizar o risco de contaminação e complicações respiratórias^(37,38)^.

### Cuidados relacionados ao cuidado da pele

A integridade da pele constitui um aspecto crítico no cuidado domiciliar de crianças com CCCs dependentes de tecnologia, especialmente em razão da presença de dispositivos médicos invasivos, da imobilidade prolongada e do uso constante de fraldas ou curativos. Lesões por pressão, dermatites irritativas, infecções fúngicas e úlceras são complicações frequentes que impactam negativamente a qualidade de vida e aumentam o risco de hospitalizações^(39)^.

A prevenção dessas complicações exige estratégias multidimensionais, incluindo higiene adequada, hidratação da pele, mudanças frequentes de decúbito, uso de superfícies de apoio e vigilância constante quanto à presença de áreas hiperemiadas ou de perda de integridade cutânea. A equipe de enfermagem, nesse contexto, desempenha papel essencial na educação dos cuidadores e na implementação de protocolos de cuidados preventivos^(40)^.

Elias *et al*.^(14)^, em seu relatório clínico, mostram que crianças com limitações de mobilidade são vulneráveis ao aparecimento de lesões de pressão e ao comprometimento da integridade da pele. Dessa forma, entre os cuidados diários, há a necessidade de mudança de decúbito regular e proteção de proeminências ósseas, particularmente em região occipital posterior, sacro, ísquio e calcanhares.

De acordo com as recomendações do Ministério da Saúde brasileiro, o manejo clínico da pesquisa de Elias *et al*.^(14)^ coloca o cuidado de troca de decúbito e prevenção de lesão de pele como uma atribuição do cuidador de crianças CCCs dependentes de tecnologia.

### Outros cuidados domiciliares diários

Outros cuidados que aparecem nesta busca bibliográfica dizem respeito à monitorização de sinais vitais, citada por Xiao *et al*.^(13)^ e Sterni *et al*.^(18)^, assim como à identificação de sinais de alerta. O monitoramento contínuo de parâmetros clínicos constitui uma dimensão essencial do cuidado domiciliar de crianças com CCCs, uma vez que permite a detecção precoce de alterações clínicas e a intervenção oportuna diante de agravos. A monitorização dos sinais vitais, como temperatura corporal, frequência cardíaca, frequência respiratória e saturação de oxigênio, deve ser realizada de maneira sistemática e documentada pelos cuidadores, com o suporte e orientação da equipe de saúde. Dispositivos como oxímetros de pulso, termômetros digitais e monitores portáteis têm sido utilizados com frequência nesse contexto^(41)^.

Xiao *et al*.^(13)^ e Elias *et al*.^(14)^ destacam como importante a identificação de sinais de agravo ou dor, cuidados emergenciais e para resolução de problemas. A identificação de sinais de alerta, tais como alterações súbitas no nível de consciência, cianose, febre persistente, convulsões, vômitos incoercíveis, dificuldade respiratória, entre outros, requer atenção constante e treinamento adequado dos familiares. A capacidade de reconhecer esses sinais e de buscar atendimento em tempo oportuno é um fator decisivo na prevenção de agravos e internações hospitalares^(42)^.

Outro aspecto relevante diz respeito à avaliação e ao manejo da dor, uma vez que muitas crianças com CCCs convivem com dor crônica associada a procedimentos, posturas mantidas, espasticidade ou doenças de base. A avaliação da dor deve ser realizada com base em escalas apropriadas à idade e ao desenvolvimento neuropsicomotor da criança, como a escala *Face, Legs, Activity, Cry, Consolability* para crianças não verbais. O manejo adequado da dor inclui tanto intervenções farmacológicas quanto medidas não farmacológicas, como massagens, posicionamento adequado e conforto ambiental^(42,43)^.

Além disso, é fundamental que os cuidadores estejam capacitados para agir diante de situações de urgência e emergência, como aspiração de vias aéreas, desconexão de dispositivos, parada respiratória ou obstrução de sonda. Protocolos de ação, listas de verificação e treinamento em primeiros socorros são estratégias que devem ser incorporadas à rotina domiciliar, promovendo maior segurança e prontidão às respostas^(25)^.

Por fim, Ribeiro *et al*.^(16)^ apontam, em seu estudo, que os cuidados cotidianos, como a higiene corporal, a troca de roupas, a manutenção da rotina de sono, o estímulo ao desenvolvimento neuropsicomotor e a atenção ao bem-estar emocional da criança, compõem o cotidiano do cuidado domiciliar. Esses cuidados, muitas vezes invisibilizados, são de extrema importância para garantir dignidade, conforto e qualidade de vida à criança e à família. Portanto, o reconhecimento da complexidade do cuidado cotidiano e o apoio sistemático às famílias devem ser premissas dos serviços de atenção domiciliar^(44-47)^.

### Limitações do estudo

O estudo apresentou limitações referentes à pouca publicação literária sobre a temática nos últimos dez anos.

### Contribuições para a área da saúde, enfermagem ou políticas públicas

Este estudo apresenta contribuição relevante e atual para a área de enfermagem pediátrica e atenção domiciliar, e traz as premissas da prática do cuidado centrado na pessoa e na família na perspectiva da saúde da criança, que reconhece a família como central na vida da criança, vê a criança no contexto de sua família e apoia os membros da família em seu papel de cuidadores. A descrição dos cuidados diários no domicílio de crianças com CCCs, aliada à parceria entre prestadores de cuidados em saúde (como profissionais de saúde e membros da família), pode colaborar na elaboração de um plano de cuidados integral e personalizado, assim como na tomada de decisão e na capacitação de cuidadores. Isso fornece ao enfermeiro o conteúdo necessário para a estruturação de treinamentos, a construção de material instrutivo (como cartilhas e *checklist* de cuidados) e a divulgação de boas práticas assistenciais voltadas para o cuidado de crianças com CCCs no domicílio.

É importante apoiar familiares cuidadores na realização de cuidados seguros e livres de danos, visando à manutenção da vida, ao desenvolvimento e ao conforto de seus filhos.

## CONCLUSÕES

O levantamento dos estudos publicados possibilitou a identificação de diversas demandas de cuidados diários necessários para as crianças com CCCs de saúde dependentes de tecnologia nos mais diferentes níveis de complexidade. O preparo do cuidador familiar se dá em um contexto hospitalar permeado de práticas educativas problematizadoras, tendo como base o diálogo e a execução de atividades essenciais realizadas e treinadas no ato, onde o enfermeiro precisa negociar saberes que não pertencem ao cotidiano de cuidado dessas famílias. Para tanto, o enfermeiro deve ser o coordenador do processo educativo, com participação ativa no preparo da família para o cuidado das crianças com CCCs no domicílio.

A complexidade das demandas de cuidados domiciliares de crianças com CCCs exige não apenas o domínio técnico dos procedimentos, mas também uma abordagem de rotinas cotidianas de vida diária sempre centrada na família que reconheça as especificidades do contexto doméstico para promoção de autonomia e empoderamento dos cuidadores. Os cuidados relacionados ao sistema respiratório, à administração de alimentos e medicamentos, às ostomias e à integridade da pele configuram áreas críticas que requerem suporte contínuo, educação permanente e articulação entre os diferentes níveis de atenção à saúde. A avaliação dos cuidados prestados pela enfermagem se faz com base na integração entre a promoção da saúde e fatores socioambientais, culturais e pessoais, que afetam o bem-estar da criança ou adolescente e de sua família.

Assumir a responsabilidade pelo cuidado e gerenciamento de crianças dependentes de tecnologia é um evento que altera a vida da maioria das famílias e cuidadores. Entretanto, o treinamento de pais e familiares como cuidadores foi relatado em todos os estudos desta revisão como muito importante e relevante para experiências de sucesso no domicílio. Essas informações podem auxiliar na proposição de um programa de treinamento para pais cuidadores de crianças dependentes de tecnologias e de longa permanência hospitalar.

## Data Availability

Os dados de pesquisa estão disponíveis no corpo do artigo.
